# Preliminary Design and Evaluation of a B-Scan OCT-Guided Needle

**DOI:** 10.3390/photonics1030260

**Published:** 2014-09-12

**Authors:** Karen M. Joos, Jin-Hui Shen

**Affiliations:** Vanderbilt Eye Institute, Vanderbilt University, Nashville, TN 37232-8808, USA

**Keywords:** optical coherence tomography, image-guided needle, forward-imaging probe, ophthalmic device, ophthalmology

## Abstract

Real-time intraoperative B-scan optical coherence tomography (OCT) visualization of intraocular tissues is a desired ophthalmic feature during retinal procedures. A novel intraocular 25-gauge B-mode forward-imaging OCT probe was combined with a 36-gauge needle into a prototype instrument. Imaging of the needle tip itself and the effects of saline injection into a gelatin phantom were performed. A combined B-scan forward-imaging OCT-needle prototype was capable of real-time-imaging of saline injection into a gelatin phantom. Additional future miniaturization may permit this instrument to be an adjunctive realtime imaging and procedure tool for vitreoretinal surgery.

## 1. Introduction

Optical coherence tomography imaging is widely used in ophthalmology clinics for diagnosing retinal disorders. External microscope-mounted OCT operating room systems have imaged retinal changes immediately following surgical manipulations [1–3]. Optical coherence tomography has been used successfully in the operating room to monitor pre- and post-procedure ophthalmic procedures including ablation of retinal lesions [4], macular hole, retinal detachment, and epiretinal membrane repairs [5–9]. However, it would be ideal to image critical surgical maneuvers in real time. A three-dimensional scanning lens optical coherence tomography intraoperative system with maximum speed of 10 volumes/s has been developed which enabled viewing in a 3 mm × 3 mm × 3 mm volume of dissecting an arteriole in an *ex vivo* bovine retina [10]. Besides being used to evaluate a patient peri-operatively, OCT has been added to cataract and anterior segment surgical systems to increase surgical precision [11–14]. Several experimental external microscope-mounted OCT systems have been developed for the posterior segment [1–3,9,15–18], although current limitations include challenges tracking constantly moving intraocular surgical instruments, and formation of absolute shadows by the metallic surgical instruments upon the underlying tissues of interest [19–23]. A 25-gauge intraocular OCT-imaging probe was developed to resolve these problems [24]. A 36-gauge needle tip was combined with the probe to permit real-time imaging of the needle tip as it touched a gelatin phantom, penetrated the gelatin, and injected saline into the gelatin phantom to form a discrete fluid pocket. A combined B-scan OCT-needle was capable of real-time imaging maneuvers without requiring the aid of a surgical microscope.

## 2. Experimental Section (Development and Testing of a B-scan OCT-guided 36-Gauge Needle)

**Figure 1:**
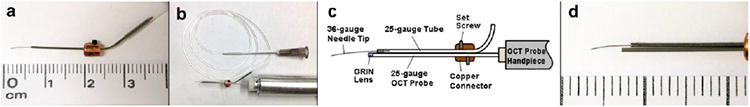
(**a**) Photograph of the 36-gauge needle enclosed within a 25-gauge tube. (**b**) Photograph of the 36-gauge needle/25-gauge tube coupled to the 25-gauge B-scan optical coherence tomography (OCT) probe. (**c**) Diagram of the 36-gauge needle enclosed within a 25-gauge tube coupled to the 25-gauge B-scan OCT probe. The tip extended 3.5 mm from the tip of the OCT imaging probe. (**d**) Magnified view of the 36-gauge needle enclosed within a 25-gauge tube coupled to the 25-gauge B-scan OCT probe.

A 36-gauge needle was placed within a 25-gauge cannula which was curved at the proximal end and attached to a 23-gauge thin-wall metal sleeve. This sleeve was attached to soft silicone tubing (1 mm outer diameter, 0.5 mm internal diameter) to permit the injection of fluid. A 3.5 mm-diameter and 4 mm-long copper connector with 2 drilled 25-gauge channels was attached to the 25-gauge cannula with a side 0-80 set screw to enable coupling to the 25-gauge disposable tip cannula of a forward-imaging B-scan OCT probe. The connector permitted adjustment of the needle tip to allow optimization of B-scan OCT imaging of the needle tip. The 36-gauge needle tip extended 3.5 mm beyond the cannula tip with a smooth curve to permit imaging of the needle tip by the forward-imaging OCT probe (Figure 1a).

The forward-imaging OCT probe consisting of a 100 mm long cylindrical body with a 12.7 mm diameter handpiece was described previously [24]. Briefly, a disposable 25-gauge extra-thin-wall probe tip extended beyond the end of the handpiece. An electromagnetic controller is embedded within the handpiece to drive the 125 μm single-mode fiber optic actuator within the 25-gauge probe tip. A sealed 0.35 mm diameter, 0.5 mm thick, < ¼ pitch GRIN lens (Go!Foton, Somerset, NJ, USA) within the probe tip protected the fiber scanner and focused the scanning beam 3 to 4 mm distant. The OCT engine was a spectral-domain optical coherence tomography (SDOCT) system (870 nm, Bioptigen, Inc. Durham, NC, USA) which produced 2000 A-scan lines per B-scan image at a frequency of 5 Hz with the fiber optic oscillations matched to this frequency. The axial resolution was 4-6 μm and the lateral resolution was 25-35 μm [24].

The B-scan OCT probe was then mechanically coupled to the 36-gauge needle/25-gauge cannula through the second 25-gauge channel in the copper connector as illustrated in Figure 1b-d. Positioning of the relationship between the OCT probe cannula and the needle tip was adjusted with the set screws. The curvature of the needle tip was adjusted to permit visibility of the tip in the real-time OCT images. Real-time imaging of the needle tip as it touched the gelatin phantom, penetrated the gelatin, and injected saline into the gelatin phantom was performed. The gelatin was formed with 20 g Knox Gelatine powder (Kraft Foods Global, Inc., Northfield, IL, USA) and 60 g Coffee-mate (Nestle USA, Glendale, CA, USA) in 250 mL hot water poured into petri dishes. A 0.2 to 0.5 mm thick gelatin layer was added later to form the top layer of the phantom.

## 3. Results and Discussion

A first-generation combined forward-imaging B-scan OCT-guided needle prototype was successfully developed. This instrument demonstrated real-time imaging at the needle tip.

### 3.1. Design of the Combined OCT Probe and Needle

**Figure 2:**
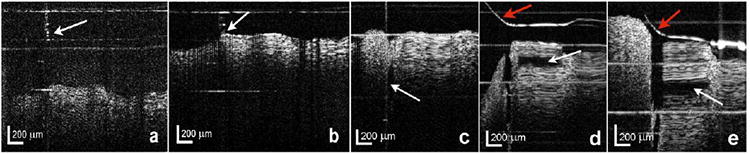
(**a**) Frame from a real-time OCT video of the 36-gauge needle tip (arrow). (**b**) Frame from a real-time OCT video of the 36-gauge needle tip (arrow) touching a gelatin phantom. (**c**) Frame from a real-time OCT video of the needle inserted into the gelatin phantom with early deposition of saline (arrow). (**d**) Frame from a real-time OCT video of separation of the upper gelatin layer with saline deposition (white arrow) through the 36-gauge needle. The bright line (red arrow) is the refluxed saline meniscus with expected upward curvature near the needle's shaft. (**e**) Frame from a real-time OCT video of another separation trial of the upper gelatin layer (white arrow) with saline injection. Again, the bright line (red arrow) is the refluxed saline meniscus with expected upward curvature near the needle's shaft.

The miniature forward-imaging B-scan OCT probe without tools and the OCT scanner was previously described in detail [24]. Briefly, the focus point of the probe is approximately 3.5 mm distance, but has a working distance range of 3 to 4 mm. The beam waist was measured as 25 μm at 3 mm and 35 μm at 4 mm distant [24]. The SDOCT SLD central wavelength was 870 nm with spectral width of 90 nm. The optical output power was 700 μW with an axial resolution of 4–6 μm (VHR SDOCT, Bioptigen, Inc, N.C., USA). A 36-gauge needle was chosen to limit the size of the puncture hole. Curvature of the needle tip permitted alignment of the needle tip with the OCT scanning beam. The needle tip was 3.5 mm distal to the OCT probe tip to coincide with the optimal OCT focal plane. The copper connector (Figure 1a-c) allowed additional adjustment of the relationship of the forward-imaging OCT probe and the needle tip to optimize the needle's image on the monitor. A frame from an unprocessed real-time video in Figure 2a shows the needle tip in the air prior to the experimental testing. After optimization, the relationship between the OCT probe and the needle tip remained constant during the evaluation.

### 3.2. Evaluation of the Combined OCT Probe and Needle

The 36-gauge needle tip is visible touching the surface of the gelatin phantom in Figure 2b. The needle tip penetrated the gelatin in Figure 2c without the aid of a surgical microscope and an initial deposition of saline is visible in this OCT image. Then obvious elevation of the thin overlying gelatin layer with a discrete saline pocket is apparent on the monitor in real time during the injection as illustrated in a single unprocessed frame from the video in Figure 2d. Another injection trial is shown in Figure 2e with similar localized elevation of the thin gelatin layer by the saline. The OCT probe system does comply with the specific technical meaning of “real-time” with no perceivable delay of the displayed images as the needle is inserted into the gelatin and the fluid is injected. No image averaging or post-image processing occurred. The resulting images are not as high quality as static images which undergo processing after acquisition, but are adequate for monitoring a real-time action. The images in Figure 2 were extracted from real-time videos and are single frames without post-acquisition processing. The initial prototype 25-gauge forward-imaging B-scan OCT probe combined with a 36-gauge needle tip was designed and developed for pre-clinical retinal surgery. This probe has an internal scanning system so it can be held steady to produce a two-dimensional B-scan image, unlike common-path A-scan probes [25,26]. Although the combined OCT-needle prototype's diameter of 0.51 mm by 1.1 mm was a 19-gauge size usable in the past for vitrectomy surgeries, it is too large for passage through the 23-gauge ports commonly used in current vitrectomy procedures. Future work will include designing and refining a smaller diameter combined probe which will pass through the 23-gauge vitrectomy ports. The 36-gauge (0.10 mm) needle will be directly attached to the shaft of the disposable 25-gauge OCT probe tip (0.51 mm) which will make it a maximum 0.61 mm + 0.03 mm weld = 0.64 mm in diameter which will pass through a 23-gauge (accommodating 0.64 mm diameter instruments) vitrectomy port. In addition, enhanced image quality would be expected with a faster imaging OCT engine. The probe's fiber has been tested to scan at 20 Hz without problems. The probe is adjustable to match the acquisition speeds of different OCT systems.

## 4. Conclusions

Real-time intraocular B-scan optical coherence tomography (OCT) visualization and feedback of surgical maneuvers is a desired feature. An intraocular 25-gauge B-scan forward-imaging probe coupled with a 36-gauge needle tip was developed. A combined B-scan OCT-needle was capable of real-time performance and imaging of saline injection into a gelatin phantom to form a discrete fluid pocket without requiring the aid of a surgical microscope. In the same manner as an intraocular endoscope, the OCT probe will bypass corneal and lenticular opacities for improved imaging. With future size reduction, the forward-imaging B-scan OCT-needle probe may become an integral tool during vitreoretinal surgical procedures to overcome some issues associated with external surgical microscope-mounted OCT systems.
